# Laparoscopic Ovarian Transposition and Ovariopexy for Fertility Preservation in Patients Treated with Pelvic Radiotherapy with or without Chemotherapy

**Published:** 2020-01-24

**Authors:** L Turkgeldi, A Cutner, E Turkgeldi, A Al Chami, A Cassoni, N Macdonald, T Mould, A Nichol, A Olaitan, E Saridogan

**Affiliations:** University College London Hospital, Women’s Health Division, 250 Euston Road, London NW1 2PG, United Kingdom;; Current address: ‘Fulya Bahceci IVF Centre’, Istanbul, Turkey;; Current address: Koc University Hospital, Istanbul, Turkey;; University College London Hospital, Cancer Division, 250 Euston Road, London NW1 2PG, United Kingdom.

**Keywords:** Ovarian transposition, ovariopexy, pelvic radiation, fertility preservation, cancer

## Abstract

**Background:**

Preservation of fertility in cancer patients of reproductive age is a concern for both the patient and the clinician. In this study, we aimed to study the effectiveness of laparoscopic ovarian transposition or ovariopexy in preserving ovarian function in women undergoing pelvic radiotherapy with or without chemotherapy for pelvic tumours.

**Methods:**

The records of patients who underwent laparoscopic ovarian transposition or ovariopexy prior to pelvic radiation therapy between 2002 and 2018 were reviewed retrospectively.

**Results:**

Thirty-nine women or adolescent girls with a diagnosis of cervical cancer (n=15), Hodgkin’s lymphoma (n=6) or other types of pelvic tumours (n=18) were included in the study. The majority of patients had bilateral (n=25) or unilateral (n=8) ovarian transposition prior to radiotherapy. Nine out of 10 (90%) patients with soft tissue tumors, Ewing sarcoma or ependymoma, five out of seven (71.4%) patients with Hodgkin’s lymphoma, two patients (100%) with rectal and anal cancer, and six out of 15 (40%) with cervical cancer retained ovarian function. Patients with cervical cancer, those who received concomitant chemotherapy and those older than 30 years were more likely to experience ovarian failure. Five patients conceived spontaneously and two women had four live births.

**Conclusion:**

Laparoscopic repositioning of the ovaries out of the radiation field in order to protect ovarian function in patients receiving radiotherapy appears to be effective in the majority of patients. The procedure seems safe and should be considered either as a sole procedure or in association with other fertility preservation methods prior to pelvic radiotherapy.

## Introduction

The current clinical practice and research regarding fertility preservation prior to treatment for cancer are focused on gamete/embryo freezing and ovarian tissue cryopreservation. Whilst this approach has enabled patients to have children in the future, it does not necessarily help to preserve hormone secretion and warrants the use of assisted reproductive technology for conception. Furthermore, in some cases there may either be insufficient time to freeze eggs/embryos or access to ovarian tissue freezing may not be available. Repositioning of the ovaries prior to pelvic radiotherapy may be an alternative option when chemotherapy is either not required or is not expected to cause total ovarian failure.

Ovarian transposition (OT) and ovariopexy (or oophoropexy, OP) are surgical procedures performed to reposition the ovaries out of the radiation field in order to protect ovarian function in patients receiving radiotherapy. Originally performed by laparotomy, OT and OP are currently carried out laparoscopically before initiation of radiotherapy (Visvanathan et al., 2003; Falcone and Bedaiwy, 2005). Ovarian transposition with robotic surgery has also been described in the literature (Iavazzo et al., 2013).

The reported success rates of ovarian transposition in the prevention of ovarian failure range from 16% to 90% (Tulandi and Al-Took, 1998; Sonmezer and Oktay, 2004; Ronn and Holzer, 2013; Mossa et al., 2015). The age of the patient population, the dose and route of radiotherapy, the shielding of ovaries, ability to prevent scatter radiation and the presence of chemotherapy may all account for the variation among the results of these studies.

In this report, we aimed to present our own experience with laparoscopic ovarian transposition/ ovariopexy and determine factors affecting the rates of preservation of ovarian function in patients who received pelvic radiation with or without chemotherapy.

## Materials and methods

### Study population

The files of 39 patients with various malignancies who underwent laparoscopic ovarian transposition/ ovariopexy prior to pelvic radiation therapy at the University College London Hospital between 2002 and 2018 were reviewed retrospectively. Data regarding the age at diagnosis, type of transposition/ ovariopexy, radiotherapy, chemotherapy, oncological outcome, hormonal status and menstrual history following therapy were recorded. The mean age at which OT/OP was performed was 25.3 years (range, 13 to 35).

Thirty-four out of 39 patients were included in the final analysis. The remaining five patients were excluded as three patients died and in two patients it was not possible to retrieve data related to menstruation, hormone profile, or conception.

### Radiotherapy

Twenty-eight patients received chemotherapy. Among them, 11 were treated with cisplatin, six with OEPAx+ COPP, two with VIDE, four with carboplatin and paclitaxel followed by cisplatin, two with the IVA regimen, one with MAP regimen, one with gapecitabine, and one with 5FU regimen. Two patients received doxorubicin following local recurrence of disease. Two patients received tamoxifene and four patients did not receive chemotherapy or hormonal therapy. GnRH analogues were not administered to any of the patients.

### Surgical Procedure

The OT or OP procedures were performed just prior to radiotherapy. The choice between OT and OP depended on whether it was possible to move the ovary out of the radiation field whilst maintaining its blood supply from both the ovarian ligament and infundibulopelvic ligament. When this was possible an OP procedure was performed to pull the ovary out of the radiation field whilst retaining its ligaments. When the radiation field covered the entire hemipelvis or whole pelvis-hence an OP procedure was not sufficient- an OT procedure was chosen to move the ovary/ovaries out of the pelvis into the upper abdomen.

**Figure 1:**
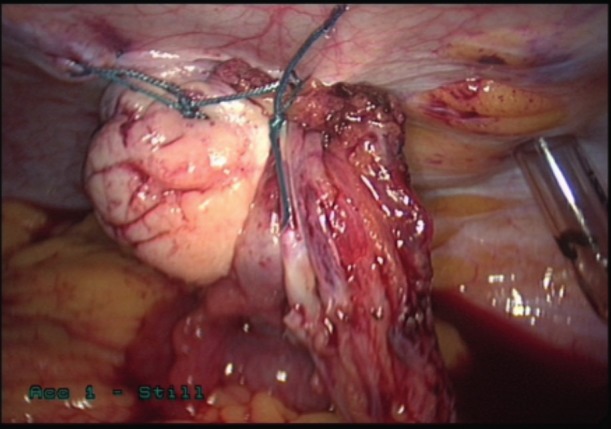
Left ovarian transposition. The ovary has been sutured to the left anterolateral abdominal wall below the costal margin.

In OT procedures the ovary was detached from the uterus by dividing the ovarian ligament, whilst maintaining the infundibulopelvic ligament and moved out of the pelvis into the upper abdomen outside the radiation field. The ovarian ligaments and fallopian tubes were divided and separated from the uterus, using a harmonic scalpel. In more recent cases, the fallopian tubes were left attached to the uterus, and only the ovaries were transposed. An ovarian pedicle was formed by incising the peritoneum parallel to the infundibulopelvic ligaments until an adequate length was achieved to reach the costal margin without tension. The mobilized ovaries were sutured with two non- absorbable sutures to the peritoneum on the anterolateral abdominal wall below the lowest costal margin. Each transposed ovary was tagged with two radioopaque clips so that they could be identified on the planning CT scan, the dose minimised and accurately recorded (Figure 1).

**Figure 2:**
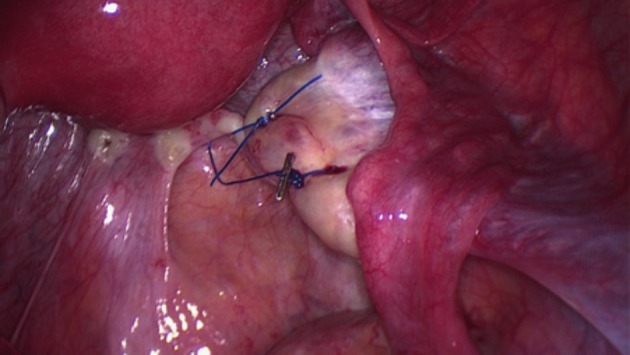
Right medial ovariopexy. The right ovary has been sutured onto the right uterosacral ligament. Further medial move can be achieved by an additional suture to pull the ovary onto the posterior uterine wall, if required.

**Figure 3:**
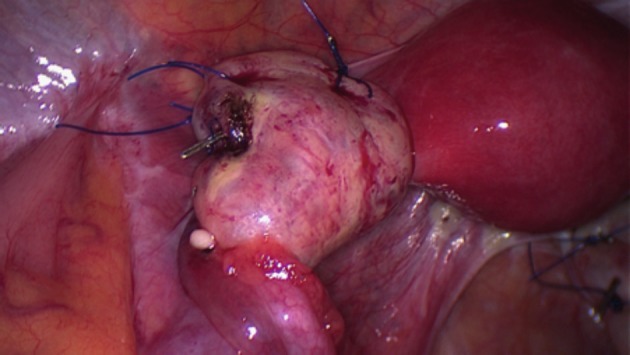
Left anterolateral ovariopexy. The left ovary has been moved laterally by suturing to the left round and lateral umbilical (obliterated umbilical artery) ligaments.

In OP procedures, both the ovarian and infunbidulopelvic ligaments were maintained and the ovary was moved out of the radiation field by suturing it onto a supporting structure such as the uterosacral ligament (Figure 2), posterior uterine wall, obliterated umbilical artery, iliopectineal ligament or round ligament (Figure 3). The site of OT/OP was determined according to the anticipated field of radiation. When the ovaries were within or adjacent to the target volume, they were transposed out of the pelvis (OT), those on the opposite side of the pelvis and at risk from a single incident field or scattered radiation were placed as far away as possible, while retaining vascular and fallopian tube connections (OP). Five patients underwent unilateral OT and contralateral OP. For one patient diagnosed with rectal cancer, a concurrent uterine suspension to the anterior abdominal wall was also performed.

Laparoscopic release of ovaries was performed in eight patients who underwent OP, 1-13 months after completion of radiotherapy. We did not attempt to release ovaries after OT, in order to avoid injury to the blood supply.

### Assessment of ovarian function

The presence of ovarian function was determined by assessing the presence or absence of menstruation without hormone replacement, occurrence of pregnancy and measurements of serum FSH, LH and oestradiol (E2) levels. The duration of follow up ranged from one to 14 years.

### Statistical analysis

Age of included patients is given in years and proportions are expressed as percentages. No comparisons were made between groups.

### Ethical approval

Ethical approval was not required as the project was considered to be solely service evaluation. Such projects do not require ethical review by an NHS or Social Care Research Ethics Committee or management permission through the NHS R&D office. Under these circumstances, there was no need to submit applications to the NHS Research Ethics Committee (REC) or NHS/HSC R&D office (www.hra.nhs.uk).

## Results

In the 39 patients included, the most common diagnoses were cervical cancer, lymphoma and soft tissue sarcoma (Table I). Twenty-five patients underwent bilateral OT, three underwent unilateral OT, six had unilateral or bilateral OP, and five had a unilateral OT and OP of the contralateral ovary (Table II). Twenty-two out of 34 (64.7%) patients with known outcome, retained some degree of ovarian function and the remaining 12 (35.3%) patients developed ovarian failure. All patients with cervical cancer received radiotherapy and chemotherapy and nine out of 15 (60%) became menopausal. Ten out of 11 (90.9%) patients with soft tissue tumors, Ewing sarcoma or ependymoma retained their ovarian function. Five out of the seven (71.4%) patients with Hodgkin’s lymphoma and one of the two patients with rectal and anal cancer retained their ovarian function. Of the 12 patients that experienced ovarian failure, nine (75.0%) were treated for cervical carcinoma. Five out of 7 (71.4%) patients aged 30 years or above and seven out of 27 patients (25.9%) below the age of 30 years experienced ovarian failure. All six patients who had radiotherapy without chemotherapy retained their ovarian function.

**Table I:** — Types of malignancies in 39 patients who underwent OT or OP.

CANCER TYPE	NUMBER OF PATIENTS
Cervical cancer	15
Hodgkin’s lymphoma	6
Desmoid fibromatosis	4
Ewing sarcoma	3
Osteosarcoma	2
Rhabdomyosarcoma	2
Soft tissue sarcoma	2
Anal cancer	1
Fibrosarcoma	1
Myxopapillary ependymoma	1
Non-hodgkin lymphoma	1
Rectal cancer	1
TOTAL	39

**Table II:** — Types of fertility preservation procedure.

Bilateral ovarian transposition	25
Unilateral ovarian transposition	3
Bilateral oophoropexy	5
Unilateral oophoropexy	1
Unilateral ovarian transposition + unilateral oophoropexy	5

One patient had a successful transabdominal egg collection procedure for egg freezing after completion of her rectal cancer treatment following bilateral OT procedure. Five patients conceived spontaneously following treatment for cancer.

All had reversal surgery prior to conception except one patient who had an unplanned pregnancy and was diagnosed with a missed miscarriage which was not preceded by menstruation 2 years after OT (the Fallopian tubes were in their original location), followed by chemotherapy and radiotherapy. Ultrasound showed that the transposed ovaries were still under the costal margins and had not migrated back into the pelvis. One patient had a spontaneous miscarriage. Another had three spontaneous pregnancies and live births and was recently diagnosed with recurrence of fibromatosis 10 years after her primary diagnosis. Another patient delivered four years after surgery and radiotherapy. One patient had a voluntary termination one year following radiotherapy.

None of the patients developed clinically diagnosed ovarian metastasis. Two patients with soft tissue tumors received doxorubicin due to local recurrence (two and three years after initial therapy) and one of them died due to doxorubicin induced cardiotoxicity. One patient is currently undergoing chemotherapy for pulmonary metastasis of Ewing’s sarcoma, another patient died after recurrence of tumour six years after primary diagnosis and a third patient died because of pulmonary metastasis one year after primary diagnosis. A simple asymptomatic ovarian cyst was detected in the follow up CT of one patient. One patient treated for cervical cancer developed small bowel obstruction thought to be due to radiation induced strictures, four months following radiotherapy.

Of the seven patients who underwent laparoscopic release of the ovaries after completion of their radiotherapy, two patients were found to have an ovary that had migrated back to its original position.

## Discussion

Improved survival rates after cancer treatment have increased expectations from treatment and fertility preservation has now become an important part of the discussion prior to treatment of cancer. Preserving the ovarian function following pelvic radiotherapy gives a patient the ability to conceive naturally in the future, it also has the advantage of continuing hormone secretion and avoiding the need for hormone replacement therapy.

OT was first described in 1958 in patients irradiated as treatment for cervical cancer and was performed at the time of radical hysterectomy (McCall et al., 1958). Since then, OT has been undertaken on patients with various types of malignancies including Hodgkin’s lymphoma, vaginal cancer, rectal cancer, soft tissue sarcomas, and central nervous system (CNS) tumours (Mitchell et al., 2007; Elizur et al., 2009; Irtan et al., 2013; Gubbala et al., 2014).

Ovaries are very radiosensitive and radiation doses administered for the treatment of cancers of the cervix, endometrium, rectum, bladder and pelvic lymphomas range from 30 to 60 Gy, which will uniformly induce ovarian failure (Moawad et al., 2017). By transposing the ovaries out of the field of radiation, the ovarian dose is reduced to 5-10% of that of nontransposed ovaries (Georgescu et al., 2008).

In our study, 67.6% of patients retained ovarian function. In patients with soft tissue cancers Ewing sarcoma and ependymoma the rate of ovarian preservation was 90%. This result is quite encouraging. There are no studies involving large numbers of the afore-mentioned malignancies regarding the efficacy of OT/OP. Morice et al. (1998) reported on 24 patients among whom 2 had soft tissue sarcoma, one had Ewing sarcoma and one had ependymoma. Out of these four patients, only one patient with Ewing sarcoma was found to be menopausal following cancer treatment.

In patients with Hodgkin’s lymphoma, the ovarian preservation rate was 71.4%. This is in accordance with previous studies. Terenziani et al. (2009) reported 14 pregnancies in 11 patients with Hodgkin’s lymphoma undergoing OT at a mean age of 13 years. Williams et al. (1999) reported an overall 50% rate of ovarian preservation in adult patients with Hodgkin’s lymphoma. They concluded that nearly all women treated with radiation alone or minimal chemotherapy (0-2 cycles) would retain ovarian function and fertility; whereas those with advanced disease receiving radiotherapy with multiple courses of chemotherapy (> 2 cyles) would suffer permanent loss of ovarian function. Haie-Meder et al. (1993) concluded that MOPP chemotherapy was a prognostic factor in the development of ovarian failure. In our series, six out of seven patients with Hodgkin’s lymphoma received six cycles of chemotherapy with the OEPA regime and two out of seven developed ovarian failure.

In those patients with cervical cancer, the ovarian function preservation rate was 40%. This disappointing rate was lower than what has been reported in previous studies for women with OT in cervical cancer. However, almost all of these studies were performed on patients who had not received chemotherapy. Feeney et al. (1995) and Chambers et al. (1990) reported the rate of ovarian preservation in patients with cervical cancer receiving various degrees of radiotherapy to be 50% and 71%, respectively. Morice et al. (2000) reported a 90% rate of ovarian preservation in patients treated with brachytherapy only and a 60% rate in those treated with external radiation therapy plus brachytherapy. Only a very small number of studies are available involving OT in patients with cervical cancer treated with both radiotherapy and chemotherapy. Huang et al. (2007) reported on 14 patients aged between 30 and 42 years, 12 of whom had cervical cancer. All 12 cervical cancer patients received concurrent chemoradiation with cisplatin. A similar ovarian function preservation rate of 50% was achieved in this study when compared with our series. Patients diagnosed with locally advanced cervical cancer at the UCLH are offered radiotherapy with concurrent cisplatin chemotherapy as standard. In addition, eligible patients are offered inclusion in the INTERLACE randomised study of carboplatin and paclitaxel chemotherapy prior to chemoradiotherapy. The addition of chemotherapy to radiotherapy offers an improvement in long- term survival but is likely to carry an increased risk of ovarian failure. Pre-treatment egg or embryo freezing should be considered but usually causes a delay in starting treatment and may increase the risk of tumour progression.

Radiotherapy is known to have a more detrimental effect on ovarian reserve when administered together with systemic chemotherapy (Moawad et al., 2017). Utilization of a combination of fertility preservation methods may be more effective in preserving future ovarian function than performing OP/OT alone in patients receiving both radiotherapy and chemotherapy (Donnez and Dolmans, 2013; Gavrilova-Jordan et al., 2015). Oocyte cryopreservation is now considered as an effective and established method of fertility preservation due to the recent implementation of novel vitrificaton techniques for the freezing of oocytes (Domingo and Garcia-Velasco, 2016; Levi-Setti et al., 2016). Ovarian tissue biopsy at the time of OT/OP and cryopreservation may be carried out for future reimplantation of ovarian tissue or in vitro maturation of primordial follicles to metaphase-II oocytes (Donnez and Bassil, 1998; von Wolff et al., 2011). Since primordial follicles are known to be more resistant to radiation and chemotherapy induced injury, GnRH analogues may be used to suppress the ovaries to simulate a prepubertal state prior to chemotherapy (Blumenfeld and Evron, 2015). Although the latter two approaches are still considered experimental, they may be offered to those patients undergoing OT/OP who cannot afford to postpone treatment for 2-3 weeks for the retrieval of oocytes (Practice Committee of American Society for Reproductive Medicine, 2013).

The success of ovarian transposition is shown to depend on several factors including the radiation dose, age at the time of radiation exposure, extent of radiation field and the presence of chemotherapy (Haie-Meder et al., 1993; Huang et al., 2007; Wo and Viswanathan, 2009; Hwang et al., 2012). Lushbaugh and Casarett (1976) suggested that the dose received by the ovaries which can induce complete menopause ranges from 3.2 to 20 Gy. It is suggested that a dose of 6 Gy would be sufficient to induce castration in patients aged more than 40 years, whereas the ovaries could withstand 20 Gy in patients younger than 20 years (Lushbaugh and Casarett, 1976; Morice et al., 1998). Chambers et al. (1990) concluded that if the dose to the ovary could be limited to 300cGy or less, the probability of retaining ovarian function would be approximately 90%. Chemotherapy is likely to lower the threshold for damage by radiation. In the present study, 71.2% of patients aged 30 years or older became menopausal whereas only 26% of those younger than 30 years were menopausal following treatment.

The final location of the transposed ovary is thought to be one of the most important factors in the successful preservation of ovarian function. A distance of 4 cm is recommended between the transposed ovary and the radiation field (van Beurden et al., 1990). Hwang et al. (2012) determined an optimum cut off distance of 1.5 cm or more above the iliac crest for the successful preservation of ovarian function after lateral ovarian transposition in cervical cancer patients receiving radiotherapy. Although the aim is to move the ovaries as far as possible from the field of radiation during the OT/OP procedure, care must be taken to avoid compromising the blood supply of the ovaries by overstretching, torsion or kinking of the infundibulopelvic ligaments. One study reported lateral ovarian transposition to be more successful than medial ovarian transposition in preserving ovarian function in patients with Hodgkin’s lymphoma (Grabenbauer et al., 1991). It is recommended to perform OT/OP immediately before radiation therapy to prevent the migration of ovaries back to their original position. In our centre, OT/OP is performed just before radiotherapy, and when possible, the ovaries are released by a second operation in those who undergo oophoropexy.

No significant pelvic adhesions were detected in any of the patients undergoing laparoscopic release of ovaries.

The reported complications of OT/OP are ovarian cyst formation, dysparunia, chronic pelvic pain, cancer metastasis to the transposed ovary, torsion of the ovarian pedicle and adhesions (Anderson et al., 1993; Husseinzadeh et al., 1994; Mahajan, 2015). Mechanical bowel obstruction is also a possibility after the OT procedures, hence some clinicians pull the ovary through a retroperitoneal tunnel to avoid this possibility. In our study, only one patient developed an ovarian cyst which was asymptomatic. Another patient with cervical cancer had to undergo laparotomy following small bowel obstruction four months after radiotherapy. This was thought to be due to radiation induced strictures. Although there are reports of cancer metastasis to transposed ovaries and laparoscopic port sites (Morice et al., 2000), the risk of these complications is considered to be negligible in patients with early cervical cancers (Clough et al., 1996; Bisharah and Tulandi, 2003). Nevertheless, because a higher risk of ovarian metastasis has been demonstrated in those with adenocarcinoma histology rather than squamous carcinoma (1.7% vs 0.5%) (Sutton et al., 1992) and in those with larger than 3 cm tumors (Tabata et al., 1987), it is recommended to avoid ovarian transposition in patients with non-squamous cancers of the cervix or bulky tumors (Morice et al., 2000).

Although OT/OP procedures have been shown to be effective in preserving ovarian endocrine function and increasing the overall life quality in cancer survivors, there are only a few reports on their ability to preserve fertility and pregnancy outcomes. Spontaneous pregnancies in patients with intact fallopian tubes after OP/OT have been reported in the literature (Kurt et al., 2007; Terenziani et al., 2009). As mentioned previously, Terenziani et al. (2009) reported 12 deliveries and 3 miscarriages in 11 patients treated for Hodgkin’s lymphoma over a median period of 14 years after ovarian transposition (Kurt et al., 2007; Terenziani et al., 2009). The median age at diagnosis and at the time of first pregnancy were 13 and 31 years, respectively. Tulandi and Al-Took (1998) reported a spontaneous pregnancy and delivery of a healthy baby following OT in a rectal cancer patient. Morice et al. (1998) reported 3 pregnancies in 2 patients with intact uteri treated for clear cell adenocarcinoma and in one patient with ovarian dysgerminoma after OT. In our series 5 patients who underwent OP or OT reported to conceive spontaneously. Natural pregnancies are not an option for patients with OT when the fallopian tubes are cut during surgery and the transposed ovaries are usually not transplanted back to their original location due to fear of injury to their vascular supply. Therefore assisted reproductive technologies will be necessary for those aiming to conceive. Two reports have described successful transabdominal retrieval of oocytes from transposed ovaries (Steigrad et al., 2005; Roberts et al., 2015). One of our patients had a successful transabdominal egg collection for egg freezing. The same patient conceived spontaneously after OT and was operated on after we modified the technique to leave the fallopian tubes attached to the uterus.

It is important to bear in mind that even though ovarian preservation may be achieved, the uterus may be adversely affected in those receiving pelvic radiotherapy. Uterine radiation has been associated with uterine fibrosis and decreased vascularity leading to implantation failure and pregnancy complications including spontaneous abortions, intrauterine growth retardation and preterm labour (Chhabra and Kutchi, 2013; Salih et al., 2015). Surrogate deliveries have been reported using oocytes obtained from patients with preserved ovaries after OT and treatment with radiotherapy or chemotherapy (Zinger et al., 2004; Agorastos et al., 2009).

OT/OP is now considered as an established method of ovarian preservation with low complication rates. However, it has been pointed out that OT/OP is an underused procedure (Han et al., 2011; Salih et al., 2015) with less than 50% of cancer patients being referred to fertility specialists for a discussion of fertility sparing methods prior to cancer treatment (Forman et al., 2009). It is important that oncologists are in close contact with fertility specialists for cancer patients to inform them about their fertility options and to increase their quality of life after treatment. In our centre OT/OP is generally offered to every patient suitable for operation taking into consideration the age at time of diagnosis, type of cancer, possibility for egg/embryo preservation and the need for emergency surgery.

In conclusion, based on the results of our study, OT/OP seems a safe and effective procedure in certain settings. Future fertility may not be guaranteed, however, there is a good possibility in preserving ovarian function. It is expected to be most effective when performed below the age of 30 years, and in those patients not receiving concurrent chemoradiation for cervical cancer. Although the success rate is relatively low in cervical cancer patients treated with concurrent chemoradiation, it can still be offered to those patients requesting ovarian preservation, provided they are informed of the risk of ovarian failure. In the presence of risk factors which indicate a high likelihood of ovarian failure, consideration should be given to additional egg, embryo or ovarian tissue freezing.
